# Vector analysis as a fast and easy method to compare gene expression responses between different experimental backgrounds

**DOI:** 10.1186/1471-2105-6-181

**Published:** 2005-07-19

**Authors:** Rainer Breitling, Patrick Armengaud, Anna Amtmann

**Affiliations:** 1 Molecular Plant Science Group, Institute of Biomedical and Life Sciences, University of Glasgow, Glasgow G12 8QQ, UK; 2 Bioinformatics Research Centre, Department of Computing Science, University of Glasgow, Glasgow G12 8QQ, UK

## Abstract

**Background:**

Gene expression studies increasingly compare expression responses between different experimental backgrounds (genetic, physiological, or phylogenetic). By focusing on dynamic responses rather than a direct comparison of static expression levels, this type of study allows a finer dissection of primary and secondary regulatory effects in the various backgrounds. Usually, results of such experiments are presented in the form of Venn diagrams, which are intuitive and visually appealing, but lack a statistical foundation.

**Results:**

Here we introduce Vector Analysis (VA) as a simple, yet principled, approach to comparing expression responses in different experimental backgrounds. VA enables the automatic assignment of genes to response prototypes and provides statistical significance estimates to eliminate spurious response patterns. The application of VA to a real dataset, comparing nutrient starvation responses in wild type and mutant *Arabidopsis* plants, reveals that consistent patterns of expression behavior are present in the data and are reliably detected by the algorithm.

**Conclusion:**

Vector analysis is a flexible, easy-to-use technique to compare gene expression patterns in different experimental backgrounds. It compares favorably with the classical Venn diagram approach and can be implemented manually using spreadsheets, such as Excel, or automatically by using the supplied software.

## Background

Large-scale gene expression measurements by microarray technology are used to compare mRNA levels in different experimental or biological conditions [1]. However, in an increasing number of cases, it seems far more relevant to compare differences in expression *responses*, rather than static expression *levels*. Perhaps the most common situation involves the comparison between a wild type and a mutant organism. Here, the mRNA profile in any condition will differ between the two genetic backgrounds, but these differences will be a complex combination of the primary effect of the mutation and secondary effects of various kinds. E.g., the mutant may show growth defects, disease reactions, or compensating adjustments in its physiology. All of these make a direct comparison between the expression profiles problematic. In contrast, comparing how organisms of each genetic background *respond* to a common relevant stimulus can reveal regulatory mechanisms that are lost or gained by the mutation as well as shared or 'disregulated' responses. Of course, the same approach is useful for other studies comparing gene expression in distinct types of background, e.g. between cell lines, tissues, or even organisms. In each case, comparing dynamic responses can provide more biological insight than a static direct comparison of expression profiles.

Despite the importance of comparing expression responses in diverse backgrounds, accessible statistical techniques for this common analytical task are sorely lacking. Usually, genes that are differentially expressed in either background are first identified independently and then compared in the form of Venn diagrams that depict the overlap between the two sets of genes (see [2-5] for examples, and [6,7] for a mathematical introduction to Venn diagrams). This approach is very attractive because of its simplicity and immediate visualization. It is implemented in many commercial microarray analysis packages (e.g. Genespring) and has also been used as an alternative to clustering techniques to identify similarities between experimental results (Venn mapping, [8]) and to visualize general relationships among the functional annotations associated with lists of differentially expressed genes [9]. Venn diagrams, however, have a number of limitations, most importantly the arbitrariness of the initial definition of changed genes. In particular, the content of the intersection of the two gene sets ("shared responses") depends critically on the selection threshold used in the initial definition of differentially expressed genes. Another disadvantage is that differential responses in the two backgrounds are not further characterized, e.g. it is not obvious whether the difference of a gene's response between the two backgrounds is due to the "regulated/non-regulated" or "up-regulated/down-regulated" effect. More sophisticated statistical techniques have been used to approach this issue (e.g. ANOVA [10], Principle Component Analysis [11], Singular Value Decomposition [12], Linear Factor Models [13], or Integrative Correlation Analysis [14]). Each of these successfully addresses certain aspects of the problem, by reducing the dimensionality of the data or identifying consistent patterns of behavior across conditions. However, they all lack the intuitive appeal and simplicity of Venn diagram visualization. Here we present a simple alternative to Venn diagrams that is based on similar concepts but provides more flexibility and an added degree of objectivity of the results.

## Results and discussion

**Figure 1:**
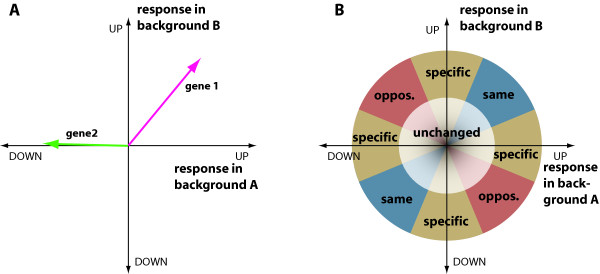
Principle of vector analysis. **(A)  **The change in expression of a gene in the two experimental backgrounds is represented by a vector. The two axes correspond to the log-fold changes in the two backgrounds. E.g., Gene 1 is strongly up-regulated in both backgrounds, while Gene 2 is specifically down-regulated in background A, but has lost this response in background B. **(B)  **The plane can be systematically subdivided into sectors corresponding to the main behavior types that are possible. In the centre, genes show very little response in either background (white). Other genes respond about the same in both backgrounds (blue sector), are specifically changed in only one background (yellow), or are regulated in opposite directions in background A and B (red).

The main underlying principle of our method (Vector Analysis, VA) is the idea that expression changes in two backgrounds can be represented by a vector in a Cartesian plane (Fig. 1A). Various sectors of the plane will correspond to various prototypical behaviors of genes: genes that respond the same in both backgrounds, genes that react in opposite directions, or genes that are changed only in one of the backgrounds (Fig. 1B). Like Venn diagrams, VA is not a method to detect differentially expressed genes, but rather a technique that arranges response patterns in an informative way for further study.

If there are replicate experiments, as is generally the case in microarray studies, we calculate the representative "average" vector *v*_*REP *_by (1) determining the individual vectors *v*^[*i*]^, where the vector *v*^[*i*] ^represents the comparison of the *i*-th pair of experiments (if there are *N* replicates in background A and *M* replicates in background B, there will be *n* = *N* × *M* pairs); (2) calculating the average length of these vectors, , where |*v*^[*i*]^| denotes the length of the vector *v*^[*i*]^; (3) calculating the sum of the unit vectors pointing in the same direction as the individual pairwise vectors, ; and finally (4) determining the representative vector by combining the length (*l*) and direction information (*v*_*SUM*_), .

The length of the vector (*l*) indicates the average strength of the response and can be used to filter out genes that show little response in either background. The direction of the vector describes which prototypical behavior comes closest to the behavior of this particular gene. To decide on the assignment of a particular gene to a response prototype, one can calculate the angle between the representative vector and the various possible prototype vectors (e.g.,  or ) as cosα = *v*_*REP*_·*v*_Prototype_/(|*v*_*REP*_||*v*_prototype_|), 0 ≤ α < 180°, where *v*_*REP*_·*v*_prototype _is the scalar product of the two vectors and |*v*_*REP*_| ≠ 0. The gene is then assigned to the prototype closest to it (minimal α).

**Figure 2:**
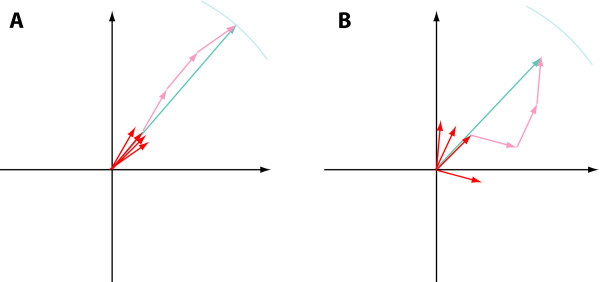
Principle of determining the consistency of the observed behavior pattern. The gene in panel **(A)  **shows highly consistent regulation among the various pairwise comparisons of replicates. Hence the corresponding unit vectors add to a long sum vector. The gene in panel **(B)  **is noisier and slightly inconsistent in its response pattern among replicates. Its vectors add to a shorter sum vector.

The length of the sum vector (|*v*_*SUM*_|) indicates the level of consistency with which the gene shows the assigned behavior type (Fig. 2). If in the individual pairwise comparisons the vectors point in widely varying directions, they will cancel out and the sum vector will be relatively short (the most probable length will approach 0 as the number of replicates increases to infinity). If, however, the behavior is fully consistent, the length of the vector will be maximal.

It is clear that the vector approach generalizes to multi-dimensional cases, i.e. to comparisons between more than two backgrounds. However, the number of possible prototype behaviors increases rapidly, as *N* = 3^*k *^- 1, where *k* is the number of dimensions.

By randomly sampling from the measured expression values and calculating the sum vector lengths for these random data (which should not show consistent behavior) one can estimate the null distribution of the sum vector length. This is done by randomly assigning the original expression values within each replicate to other genes. All consistency between replicates and, thus, between experimental backgrounds should then be lost and the resulting |*v*_*SUM*_| values will be those that are expected if no consistency is present. This can be used to assign a *p*-value to the assignment of genes to behavior prototypes (*consistency* p-*value*). This value, calculated by the procedure described above, will be a non-parametric estimate of the real *p*-value, and the exact value will vary slightly in each run of the method, unless the same random sampling is used each time.

Additional file 6 shows the results of vector analysis applied to a simulated dataset, where the response type of each gene is known [see Additional file 6]. Three replicates for each experimental background were created by drawing random expression values from normal distributions with variance 1 and a mean of 0, -2, and 2 for unchanged, down-regulated and up-regulated genes, respectively. In this small illustrative example, 87.5% of regulated genes are assigned the correct response type. The remaining genes are assigned one of the neighboring types. Genes that are *unchanged* in both conditions are also assigned to the closest response prototype, but none of these achieves a significant consistency *p*-value. Of course, in a real-world application unchanged genes would usually be filtered before applying vector analysis, because otherwise they will be assigned arbitrary angular and location values that add noise to the results. If VA is applied to genes that are not changed at all, it will always assign these genes to "incorrect" response classes, and even when the consistency *p*-value of VA is used, some of these genes will reach significance simply due to multiple testing. Therefore, VA is usually applied only to genes that are significantly changed in at least one experimental background, based on any of the standard methods for the detection of differentially expressed genes. However, the filtering does not have to be very strict and the results of VA may still yield interesting trends for borderline cases, as shown in the example below.

**Figure 3:**
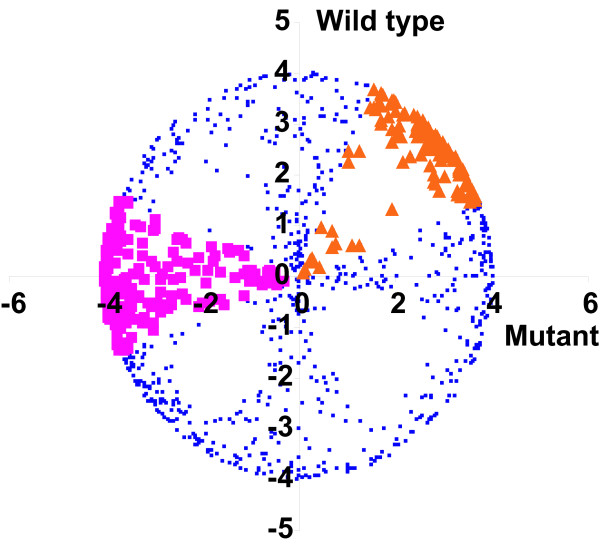
Vector analysis of gene expression responses in two genetic backgrounds in *Arabidopsis* plants. Each dot corresponds to a single sum vector from four pairwise comparisons (two replicates per background). Genes towards the periphery of the circle show the most consistent behavior among replicates. Two behavior prototypes are highlighted (corresponding to Gene 1 and 2 in Fig. 1), mutant-specific down-regulation (purple) and WT/Mutant-consistent up-regulation (orange). It can be seen that inconsistent genes (close to the center) are generally showing background-specific responses, i.e. they are enriched along the axes of the plot. Their behavior is most likely the result of spurious noise in a single replicate.

Table 1 and Fig. 3 show the results of an application of vector analysis to a real experimental dataset. The data used are a subset of a larger study examining the response of wild-type and mutant *Arabidopsis thaliana* plants to potassium starvation (Armengaud et al., unpublished data). The mutant plants (*coi1*) lack a critical component of the jasmonate signaling pathway [15], which was shown to be central for the response of plants to potassium starvation [16]. Seedling plants were grown on potassium-free agar plates for two weeks and then re-supplied with either potassium-containing or fresh deficient medium. Labeled cDNA from both conditions was prepared and analyzed on two-color whole-genome microarrays. All data were normalized by quantile normalization and log-fold changes calculated for two replicate measurements in each genetic background. A total of 1000 genes are considered in this example, which is also available as a supplementary material for further analysis.

**Table 1:** Number of genes showing the various types of prototypic behavior in two genetic backgrounds of *Arabidopsis* plants as identified by vector analysis.

Mutant specific up	162	Background-specific changes
Mutant specific down	189	
WT specific up	137	
WT specific down	122	
WT and Mutant up	133	Same-direction changes
WT and Mutant down	131	
Mutant up, WT down	54	Opposite changes
Mutant down, WT up	72	

One of the properties of this dataset is that very few genes show a strong expression response in any background. Only one out of 1000 genes has an *l*-value larger than 1 (roughly corresponding to a two-fold expression change), and only 35 genes have *l*-values larger than 0.5. Thus, a Venn analysis based on significantly changed genes would be all but impossible. The vector analysis, in contrast, identifies 32 genes with consistency *p*-values smaller than 0.01 (expected 10) and 258 genes with *p*-values smaller than 0.1 (expected 100). It thus reveals the presence of consistent response patterns even among genes with very slight absolute expression changes.

**Figure 4:**
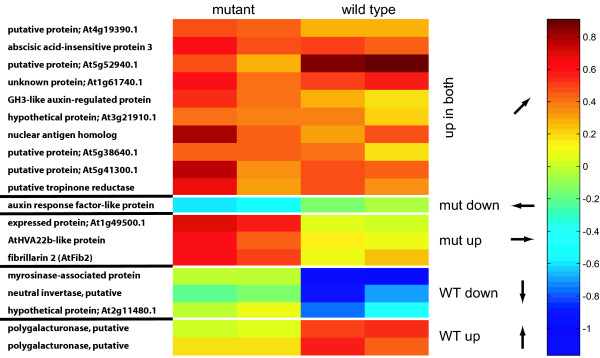
Expression change profile of the top 19 genes detected by vector analysis of starvation responses in wild type and mutant Arabidopsis plants. The genes have been filtered for vector lengths larger than 0.5 and p-values smaller than 0.1.

**Figure 5:**
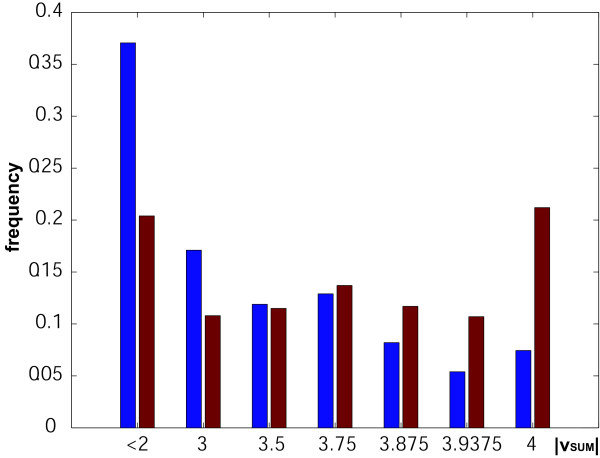
Sum vector length (|*v*_*SUM*_|) distribution for the Arabidopsis experiment and randomly permuted datasets. The real data (red) are enriched for longer sum vectors compared to random data (blue), indicating the presence of consistent response patterns.

Among the 19 most significant genes, with p-values < 0.1 and vector lengths > 0.5, more than half (10 out of 19) are up-regulated in both mutant and wild-type (Fig. 4). The remaining 9 genes show various background-specific responses. None of them shows an "opposite" response pattern, an observation that is highly significant (*p* = 0.0042). This is in agreement with the known biology of the *coi1* mutant, which will lose certain regulatory mechanisms that are important in nutrient starvation, but will not to reverse existing pathways. It is also in agreement with the overall correlation between the average expression pattern in the two backgrounds (Spearman's rank correlation r_s _= 0.310; *p* < 0.001). Importantly, the same pattern is already evident in the complete dataset (Tab. 1), where genes assigned the "opposite" prototypes are clearly depleted. The presence of a detectable signal is also confirmed by the distribution of sum vector lengths in the real data compared to randomly sampled data (Fig. 5). This indicates that even for very noisy data vector analysis is able to make meaningful assignments to response patterns.

Using the two parameters of the method (vector length = overall response intensity, and *p*-value = response pattern consistency) allows the flexible dissection of the observed expression in the two experimental backgrounds. At the same time it is possible to assign the most likely response pattern even to genes that show little absolute expression change.

In contrast to Venn diagrams, which can only be used to compare genes that are reliably identified as responsive, vector analysis assigns all genes to behavioral categories. Also note that these categories are not fixed, but can be adjusted as appropriate for any experiment, by simply changing the boundaries of the sectors. Also, genes can be sorted by their angular distance from any reference gene (or reference behavior), to generate lists that are sorted by closeness of genes to a particular response pattern.

## Conclusion

Vector analysis provides a flexible, easy-to-use, and intuitive approach to the comparison of gene expression patterns in different experimental backgrounds. While it does not supply the detailed statistical insights available by alternative classical statistics approaches such as ANOVA, it excels in terms of simplicity and straight-forward interpretation. In this respect vector analysis compares favorably with the Venn diagram technique which is currently in wide-spread use for this common and ubiquitous task, but lacks the flexibility of vector analysis, in particular for noisy data.

## Methods

For small datasets with few replicates, vector analysis is straightforward enough to be carried out manually, e.g. in Excel or OpenOffice spreadsheets. It uses only the most basic vector algebra. The Excel file in the supplementary material [see Additional file 1] demonstrates how *l*, *v*_*SUM*_, and *v*_*REP *_are calculated and used to automatically assign genes to the various response prototypes. A second sheet in the same file is used to randomly permute the experimental measurements by sorting them along a vector of random numbers, so that within each replicate (column) the original expression values are randomly assigned to new genes and all consistencies between columns are lost. The vector lengths calculated from these random data are then used in a third sheet to estimate the *p*-values associated with the observed response patterns (for details of the procedure [see Additional file 2]). For larger numbers of replicates, the manual procedure becomes quite tedious and a Perl script [see Additional file 3] is provided that performs vector analysis and *p*-value estimation automatically, taking a tab-delimited text file of log-fold changes in all replicates [see Additional file 4] as its input. The obtained results [see Additional file 5] can then be sorted, filtered and explored in various ways to dissect the details of comparative expression behavior.

## Authors' contributions

RB devised and implemented the Vector Analysis method and drafted the manuscript. PA provided the experimental data and helped with the biological interpretation of the results. AA supervised the project. All authors read and approved the final manuscript.

## Supplementary Material

Additional File 6 Output generated by vector analysis script on a set of simulated expression data with known response type for each gene. Click here for file

Additional File 1 Excel file demonstrating the manual performance of vector analysis Click here for file

Additional File 2 Word document describing the implementation of Vector Analysis in Excel and presenting the details of the equations used. Click here for file

Additional File 3 Perl script performing vector analysis Click here for file

Additional File 4 Tab-delimited text file as input file for vector analysis script Click here for file

Additional File 5 Output generated by vector analysis script Click here for file
