# Supplementary material for: Vector analysis as a fast and easy method to compare gene expression responses between different experimental backgrounds
Source: BMC Bioinformatics. 2005 Jul 19;6:181. doi: 10.1186/1471-2105-6-181 (PMC1190156; doi:10.1186/1471-2105-6-181)
Supplement: Additional File 2 — Word document describing the implementation of Vector Analysis in Excel and presenting the details of the equations used. [file 1471-2105-6-181-S2.doc]

# Description of the Excel version of Vector Analysis

The first column (A) of the supplementary file contains the gene or probe set identifiers. Columns B and C contain two replica sets of log ratios (comparing potassium-supplied and potassium-starved Arabidopsis seedlings) of the first background (label 0; *coi1* mutant), columns D and E those of the second background (label 1; wild type). Datasets with larger numbers of replicates are easily accommodated by extending the equations given below.

The Excel equations (in the second row of the datasheet) for a single gene are detailed below. In the accompanying mathematical notation, the two log ratios in the first background are $v_{0,1}$ and $v_{0,2}$, those in the second background $v_{1,1}$and $v_{1,2}$.

**Vector length** (column G). The (average) length of the vector resulting from the four possible pairwise comparisons between background 0 and background 1 responses.

$$\left|v\right|=\frac{1}{4}(\sqrt{v_{0,1}^{2}+v_{1,1}^{2}}+\sqrt{v_{0,1}^{2}+v_{1,2}^{2}}+\sqrt{v_{0,2}^{2}+v_{1,1}^{2}}+\sqrt{v_{0,2}^{2}+v_{1,2}^{2}})$$

=(1/4)*(SQRT(B2^2+D2^2)+SQRT(B2^2+E2^2)+SQRT(C2^2+D2^2)+SQRT(C2^2+E2^2))

**Vsum (component 0)** (column H). The first component of the sum vector, i.e. the sum of the unit vectors indicating the direction of the four pairwise comparison vectors.

$$v_{SUM,0}=\frac{v_{0,1}}{\sqrt{v_{0,1}^{2}+v_{1,1}^{2}}}+\frac{v_{0,1}}{\sqrt{v_{0,1}^{2}+v_{1,2}^{2}}}+\frac{v_{0,2}}{\sqrt{v_{0.2}^{2}+v_{1,1}^{2}}}+\frac{v_{0,2}}{\sqrt{v_{0,2}^{2}+v_{1,2}^{2}}}$$

=B2/SQRT(B2^2+D2^2)+B2/SQRT(B2^2+E2^2)+C2/SQRT(C2^2+D2^2)+C2/SQRT(C2^2+E2^2)

**Vsum (component 1)** (column I). The second component of the sum vector, i.e. the sum of the unit vectors indicating the direction of the four pairwise comparison vectors.

$$v_{SUM,1}=\frac{v_{1,1}}{\sqrt{v_{0,1}^{2}+v_{1,1}^{2}}}+\frac{v_{1,2}}{\sqrt{v_{0,1}^{2}+v_{1,2}^{2}}}+\frac{v_{1,1}}{\sqrt{v_{0.2}^{2}+v_{1,1}^{2}}}+\frac{v_{1,2}}{\sqrt{v_{0,2}^{2}+v_{1,2}^{2}}}$$

=D2/SQRT(B2^2+D2^2)+E2/SQRT(B2^2+E2^2)+D2/SQRT(C2^2+D2^2)+E2/SQRT(C2^2+E2^2)

**Vrep (component 0)** (column J). The first component of the representative vector, having the same direction as the sum vector and the length of the average vector.

$$v_{REP,0}=\left|v\right|\frac{v_{SUM,0}}{\left|v_{SUM}\right|}=\left|v\right|\frac{v_{SUM,0}}{\left|\sqrt{v_{SUM,0}^{2}+v_{SUM,1}^{2}}\right|}$$

=G2*H2/SQRT(H2^2+I2^2)

**Vrep (component 1)** (column K). The second component of the representative vector, having the same direction as the sum vector and the length of the average vector.

$$v_{REP,1}=\left|v\right|\frac{v_{SUM,1}}{\left|v_{SUM}\right|}=\left|v\right|\frac{v_{SUM,1}}{\left|\sqrt{v_{SUM,0}^{2}+v_{SUM,1}^{2}}\right|}$$

=G2*I2/SQRT(H2^2+I2^2)

**Vsum (length)** (column L). The length of the sum vector, which can be used to estimate the consistency of the pairwise comparisons.

$$\left|v_{SUM}\right|=\sqrt{v_{SUM,0}^{2}+v_{SUM,1}^{2}}$$

=SQRT(H2^2+I2^2)

**Angle** (column M). The angle between the summary vector and the prototype “0 specific up” in anti-clockwise direction. The arcos function determines the smallest angle between two vectors (<180 degrees in the plane), hence a conditional statement is used to assign angles between 180 and 360 degrees to vectors in the lower half of the plane.

$$\alpha=\{\begin{matrix} \arccos(\frac{v_{SUM,0}}{\left|v_{SUM}\right|}),\text{if }v_{SUM,1}>0 \\ 360^{\circ}-\arccos(\frac{v_{SUM,0}}{\left|v_{SUM}\right|}),\text{if }v_{SUM,1}\leq0 \end{matrix}$$

=IF(I2>0, (360/(2*PI()))*ACOS(H2/L2), 360-(360/(2*PI()))*ACOS(H2/L2))

**Prototype assignment** (column N). This conditional statement assigns the gene to one of the behavioral prototypes, depending on the value of the angle between the summary vector and the prototype “0 specific up”. Gene that differ from that prototype by less than 22.5 degrees in either direction (i.e. $\alpha\leq22.5\text{ or }\alpha>337.5$) are assigned to it, and accordingly for the other seven prototypes.

=IF(M2>22.5, IF(M2>67.5, IF(M2>112.5, IF(M2>157.5, IF(M2>202.5, IF(M2>247.5, IF(M2>292.5, IF(M2>337.5, "0 specific up", "0 up, 1 down"), "1 specific down"), "0 and 1 down"), "0 specific down"), "0 down, 1 up"), "1 specific up"), "0 and 1 up"), "0 specific up")

**Randomization.** The same data and equations are used in the second spreadsheet to calculate the vectors for a randomly permuted dataset. To permute the data, random numbers (RAND()) are generated in column F. Then the data are sorted by column F four times, first including columns B to F, then C to F, then D to F, and finally only E and F. Any consistency between the replicates and conditions should be lost in the resulting data.

**P-Value estimation.** The original data are copied to a third spreadsheet and the lengths of the random sum vectors are copied to column F of the same sheet, using the Paste Special option to copy only the values. The p-values are then estimated by comparing the length of each sum vector to the lengths of all randomly generated vectors.

=COUNTIF($F$2:$F$1001, ">"&L2)/1000
